# Incidence and predictors of frailty in Latin America and China: evidence from 10/66 cohort studies

**DOI:** 10.1093/eurpub/ckac129.014

**Published:** 2022-10-25

**Authors:** M Jiang, JJ Llibre Rodriguez, AL Sosa, D Acosta, IZ Jimenez-Velasquez, M Guerra, A Salas, YQ Huang, M Prince, E Albanese

**Affiliations:** 1 Institute of Public Health, Università della Svizzera Italiana, Lugano, Switzerland; 2 Facultad de Medicina Finlay-Albarran, Medical University of Havana, Havana, Cuba; 3 Laboratory of the Dementias, National Institute of Neurology and Neurosurgery of Mexico, Mexico City, Mexico; 4 Internal Medicine Department, Universidad Nacional Pedro Henríquez Ureña, Santo Domingo, Dominican Republic; 5 Internal Medicine Department, School of Medicine, University of Puerto Rico, San Juan, Puerto Rico; 6 Instituto de la Memoria Depresion y Enfermedades de Riesgo IMEDER, Lima, Peru; 7 Medicine Department, Caracas University Hospital, Caracas, Venezuela; 8 Faculty of Medicine, Universidad Central de Venezuela, Caracas, Venezuela; 9 Institute of Mental Health, Peking University, Beijing, China; 10 King's Global Health Institute, King's College London, London, UK; 11 Institute of Global Health, University of Geneva, Geneva, Switzerland

## Abstract

**Introduction:**

Evidence on the incidence and risk factors of frailty in low- and middle-income countries is very limited. We aimed to compare the incidence of frailty and explore its determinants in rural and urban areas in six Latin American countries and China.

**Methods:**

The 10/66 is a multi-site cohort study in older adults. We conducted baseline and follow-up surveys in 2003-2006, and 2007-2010. We assessed frailty using a modified Fried frailty phenotype criterion, and adjudicated frailty (yes/no) when two or more of the following indicators were present: exhaustion, low physical activity, slow gait speed, and weight loss. We excluded frail participants at baseline and calculated person-years as the time interval between baseline and follow-up for frailty-free people who were survived and reinterviewed or the midpoint of it for incident frailty cases. We used Poisson and Cox regressions to model the incidence of frailty and its risk factors.

**Results:**

We included 9,747 participants (≥65 years) for the analysis of frailty risk factors. Of whom, 8,212 were reinterviewed with an average of 4.0 years of follow-up, the incidence of frailty was lowest in Venezuela (21.9 per 1000 person-years) and rural Peru (24.3 per 1000 person-years), highest in rural Mexico (110.5 per 1000 person-years) and urban Peru (84.0 per 1000 person-years). In the overall Cox regression, we found significant prospective associations of incident frailty with living in rural areas (HR: 1.97, 95% CI: 1.69, 2.29), dementia (HR: 1.76, 95% CI: 1.42, 2.18), depression (HR: 1.69, 95% CI: 1.49, 1.93), comorbidity, female gender, older age, disability, hearing, and vision problems. Higher arm circumference was associated with a lower frailty risk (HR: 0.97, 95% CI: 0.96, 0.98).

**Conclusions:**

The incidence of frailty varied substantially in Latin America and China, and between urban and rural areas. The identified risk factors could be potential intervention targets to decrease the global burden of frailty.

**Key messages:**

• In Latin America and China, the incidence of frailty varied from 21.9 to 110.5 cases per 1000 person-years.

• We identified 9 risk factors and 1 protective factor for developing frailty, and the most relevant risk factors were living in the rural area, dementia, and depression.

